# Change in Body Mass Index and Its Impact on Incidence of Hypertension in 18–65-Year-Old Chinese Adults

**DOI:** 10.3390/ijerph13030257

**Published:** 2016-02-25

**Authors:** Qian Ren, Chang Su, Huijun Wang, Zhihong Wang, Wenwen Du, Bing Zhang

**Affiliations:** National Institute for Nutrition and Health, Chinese Center for Disease Control and Prevention, Beijing 100050, China; zheguanglushui@126.com (Q.R.); suchanglon@126.com (C.S.); wanghj128@gmail.com (H.W.); wangzh@chinacdc.cn (Z.W.); wenwendu@126.com (W.D.)

**Keywords:** body mass index, change, hypertension incidence, Chinese adults, overweight

## Abstract

Aims: This study assessed change in body mass index (BMI) and its impact on the incidence of hypertension in 18- to 65-year-old Chinese adults. Methods: Two waves of data were collected in 2006 and 2011 by the China Health and Nutrition Survey (CHNS) with samples drawn from nine provinces in China. The logistic regression model was used to examine the association between change in BMI and the incidence of hypertension, and odds ratio (OR) and 95% confident interval (95% CI) were calculated. Results: The risk of incident hypertension increased as the quartile of the BMI difference value (D-value) increased in men (OR and 95% CI for the highest quartile *vs.* the lowest quartile: 2.303, 1.560–3.401, respectively, *p* for trend < 0.001) and women (OR and 95% CI for the highest quartile *vs.* the lowest quartile: 1.745, 1.199–2.540, respectively, *p* for trend = 0.004). Compared with non-overweight subjects in 2011, the ORs of incident hypertension were all significantly higher for overweight subjects, regardless of their overweight status at baseline (*p* < 0.05). Conclusions: In conclusion, the results from this study provide unequivocal evidence that prevention of weight gain is likely to have a great impact on the incidence of hypertension in Chinese adults.

## 1. Introduction

Hypertension has been a persistent public health challenge due to its high prevalence rate [1]. The global prevalence rate of hypertension has been increasing for the past two decades [2]. Hypertension accounts for 3.7% of total disability adjusted life years (DALYS) [3].

Overweight and obesity are well-known independent risk factors for the incidence of hypertension [1,4,5,6]. The prevalence of overweight and obesity is increasing worldwide and in China [7,8]. Body mass index (BMI), waist circumference (WC), waist:hip ratio (WHR) and waist:stature ratio (WSR) are four indicators commonly used to measure the degree of obesity [4,5]. BMI appears to be sufficient for it is collected more often in nutrition and health surveys, and it is collected with universally accepted protocols compared to WC, WHR and WSR [9]. Evidence has indicated that BMI is the best predictor of incident hypertension [10].

Previous studies showed that hypertension risk increased with a higher BMI [5,11,12]. However, the association between change in BMI and the incidence of hypertension is still unclear in the Chinese population, and it may be impacted by changes in lifestyle or targeted intervention. Widely used among Western populations, the BMI cut-off value of 25 kg/m^2^ for overweight has been recommended by the World Health Organization (WHO) as an international criterion [11]. Country-specific and ethnicity-specific BMI cut-off values are suggested, for it is not appropriate to use a single cut-off value to define overweight for all populations due to the potential ethnic variation in body build and composition [13,14]. The cut-off level for overweight (24.0 kg/m^2^) has been developed using BMI for the general Chinese population by the Working Group on Obesity in China (WGOC) [15,16,17], and according to the standard of WS/T 428–2013 (China), the optimal BMI cut-off value is 24 kg/m^2^ for Chinese adults. The impact of change in overweight status based on this cut-off value on the incidence of hypertension has not been well characterized.

The aim of this study is to investigate change in BMI and its impact on the incidence of hypertension in 18- to 65-year-old Chinese adults utilizing data from the China Health and Nutrition Survey (CHNS).

## 2. Subjects and Methods

### 2.1. Study Sample

Two waves of data collected in 2006 and 2011 by the CHNS with samples drawn from nine provinces in China (Guangxi, Guizhou, Hunan, Hubei, Henan, Jiangsu, Shandong, Liaoning and Heilongjiang) are used in this study.

The CHNS is an ongoing study started in 1989, and it examined the influence of social and economic transformation among Chinese communities and society on the nutritional and health status of the Chinese population. Detailed descriptions of the study have been presented elsewhere [18]. Questionnaires and data sets can be downloaded in the following link [19]. This research was approved by the Institutional Review committees of the University of North Carolina at Chapel Hill, as well as by the National Institute for Nutrition and Health, Chinese Center for Disease Control and Prevention. All participants provided written informed consent for their participation in the survey.

Of the 5370 participants who were involved in both surveys in 2006 and 2011, whose ages were 18 to 65 in 2006, and who were non-pregnant or non-lactating women in 2006 and 2011 or who were men, 3989 (74%) had complete and plausible anthropometric measurements and measurements of blood pressure. These included, for example, a baseline BMI of 15–44 kg/m^2^; 5-year changes in height <10 cm and in BMI < 10 kg/m^2^; the difference between any two of the three measurements of systolic blood pressures (SBP) in each survey <10 mm·Hg, and the difference between any two of the three measurements of diastolic blood pressures (DBP) in each survey <10 mm·Hg [11]. Of the 3989 participants, 3215 (81%) with normal blood pressure in the survey in 2006 were included in our sample.

The exclusion of samples with implausible or extreme values in blood pressure or anthropometric measures helped us increase the estimate precision.

### 2.2. Measures

All participants underwent standardized examinations including collections of SBP and DBP. Three blood pressure measurements were taken on the right arm by trained health workers, and all of the workers followed the standardized procedure using regularly calibrated mercury sphygmomanometers with appropriate-sized cuffs. SBP was measured at the first appearance of a pulse sound (Korotkoff phase 1) and DBP was measured at the disappearance of the pulse sound (Korotkoff phase 5). Three measurements of SBP or DBP were averaged to reduce the effect of measurement error. Hypertension was defined as SBP ≥ 140 mm Hg, DBP ≥ 90 mm Hg or being diagnosed by a doctor previously [9].

Cumulative incidence was calculated by dividing new hypertension cases over the study period by the total at-risk population in 2006.

Anthropometric measures were taken after the participants had removed their shoes and heavy clothing. Height and weight were measured by the trained workers. BMI (kg/m^2^) was calculated based on weight and height which were measured by trained workers who followed standardized procedures.

The cut-off level for overweight (24.0 kg/m^2^) has been developed using BMI for the general Chinese population by the WGOC [15,16,17], and according to the standard of WS/T 428–2013 (China), the optimal BMI cut-off value is 24 kg/m^2^ for Chinese adults. Overweight was defined as BMI ≥ 24 kg/m^2^, and non-overweight was defined as BMI < 24 kg/m^2^.

Covariates such as sex, age, smoking habits, alcohol consumption, and place of residence were collected by direct interviews.

### 2.3. Statistical Analysis

The difference value (D-value) in BMI between 2006 and 2011 was calculated to evaluate the change of BMI from 2006 to 2011. D-value > 0 meant that the BMI increased from 2006 to 2011; the greater the D-value was, the more the BMI increased. D-value < 0 meant that the BMI decreased from 2006 to 2011; the lower the D-value was, the more the BMI decreased. 

Firstly, the participants were divided into two groups according to BMI at baseline: overweight and non-overweight groups. The mean and standard deviation (SD) for continuous variables and percentage for categorical variables were calculated and compared between overweight and non-overweight subjects.

The two-tail independent *t* test or chi-square test were used to test differences between overweight subjects and non-overweight subjects in the means or proportions of baseline variables such as age, SBP, DBP, BMI, place of residence, smoking habits, alcohol consumption and incidence of hypertension.

The logistic regression model was used to examine the association between change in BMI and incidence of hypertension, odds ratio (OR) and 95% confident interval (95% CI) were calculated. All potential confounding factors in the logistic regression analyses were collected at baseline. Models were initially adjusted for the baseline age, BMI, SBP and DBP (model 1). To observe the influence of lifestyle factors, models were additionally adjusted for smoking habits, alcohol consumption and place of residence at baseline (model 2). The incidence of hypertension stratified by overweight status in 2006 and 2011 was investigated by logistic regression analysis. Additionally, the association between change in SBP and DBP and change in overweight status between 2006 and 2011 was investigated by multivariable linear regression analysis. All reported *p*-values were two-tailed, and those less than 0.05 were considered statistically significant.

All statistical analyses were conducted using SAS 9.2 (SAS Institute, Cary, NC, USA).

### 2.4. Ethics Statement

This research has been approved by the Institutional Review committees of the University of North Carolina at Chapel Hill and the National Institute for Nutrition and Health, Chinese Center for Disease Control and Prevention (Ethical Review No: 201524). All participants gave written informed consent for their participation in the survey.

## 3. Results

In total, 3215 participants (1441 men and 1774 women) were studied, including 606 (18.8%) subjects (312 men and 294 women) who developed hypertension in 2011. At baseline, there were 974 non-overweight and 467 overweight men, and 1146 non-overweight and 628 overweight women. In 2011, there were 845 non-overweight and 596 overweight men, and 1030 non-overweight and 744 overweight women.

The baseline characteristics of participants stratified by sex are shown in Table 1. At baseline, the mean SBP, DBP and BMI were higher among overweight men compared with non-overweight men (*p* < 0.05). The levels of age, SBP, DBP and BMI were higher among overweight women compared with non-overweight women (*p* < 0.05). The proportions of subjects who live in urban areas were higher in overweight men than in non-overweight men.

Table 2 shows the association between incidence of hypertension and the BMI D-value. Compared with men with a BMI reduction of 0.4783 kg/m^2^ or more (*i.e.*, the lowest quartile of the BMI D-value), men with a mild reduction or gain (the second quartile of the BMI D-value of −0.4783 to 0.4920 kg/m^2^) had an increased risk for developing hypertension (OR and 95% CI: 1.448, 0.979–2.141, respectively) (model 2, Table 2). The risk for the third BMI D-value quartile rose to 1.138 (95% CI, 0.764–1.696). The risk for the highest BMI D-value quartile rose to 2.303 (95% CI, 1.560–3.401). Compared with women with a BMI reduction of 0.5953 kg/m^2^ or more (*i.e.*, the lowest quartile of the BMI D-value), women with a mild reduction or gain (the second quartile of the BMI D-value of −0.5953 to 0.4732 kg/m^2^) had an increased risk for developing hypertension (OR and 95% CI: 1.095, 0.743–1.613, respectively). The risk for the third BMI D-value quartile rose to 1.152 (95% CI, 0.778–1.706). The risk for the highest BMI D-value quartile rose to 1.745 (95% CI, 1.199–2.540). There was a significant trend of increased incidence of hypertension with an increase in the quartile of the BMI D-value in men (*p* for trend < 0.001) and women (*p* for trend = 0.004).

The participants were further stratified by overweight status in 2006 and in 2011. Compared with non-overweight subjects in 2011, overweight was associated with a 1.469-fold and 1.580-fold increase in the risk of incident hypertension in non-overweight subjects and overweight subjects at baseline, after adjustment for important covariates (*p* < 0.05) (Table 3).

Overweight subjects in 2011 had a significantly higher increase in SBP and DBP compared with non-overweight subjects in 2011 regardless of their overweight status at baseline, after adjustment for important covariates (*p* < 0.05) (Table 4).

## 4. Discussion

We measured change in BMI and its impact on the incidence of hypertension, and the association between changes in overweight status based on the BMI cut-off value recommended by the WGOC and according to the standard of WS/T 428–2013 (China) and the incidence of hypertension in 18- to 65-year-old Chinese adults. The results of this study showed that increased BMI was positively associated with the incidence of hypertension after adjustment for some important potential confounding factors, both in men and women. Our findings implied that change in BMI could affect the change of blood pressure (BP), and reducing BMI by modifying lifestyle could prevent and control incidence of hypertension.

The association between BMI at baseline and the incidence of hypertension during follow-up has been analyzed in previous studies [5,11,12]. Overweight status might change due to lifestyle modification. The change of BMI may be mainly caused by lifestyle change or targeted intervention. Several studies have reported the impact of such a change on hypertension risk [1,20,21,22,23,24]. A meta-analysis of randomized controlled trials showed that weight loss was very important for the prevention and treatment of hypertension [20]. BMI changes were independently associated with blood pressure changes for men and women in Tromso’s Study in Norway [21]. Weight loss was found to reduce the risk for hypertension in Huang’s study in a cohort of US females [22]. A prospective study in Japan found that weight gain increased the risk of developing hypertension among Japanese men and women [23]. Change in BMI and its impact on the incidence of hypertension in the Chinese should be detected because there are potential ethnic variations in body composition and build, and potential ethnic variations in the health risks associated with obesity among populations [14]. However, there is controversy regarding whether change in BMI impacts the incidence of hypertension in the Chinese [1,24]. In Chen’s study in a Taiwan community in China, BMI gain is associated with the risk of developing hypertension in men, but the risk in women is influenced by their menopausal status [1]. In Luo’s study in Jiangsu province in China, change in BMI was associated with the incidence of hypertension in both men and women regardless of the women’s menopausal status [24]. Furthermore, abnormal BMI (overweight or obesity) was defined as BMI ≥ 25 kg/m^2^ in Luo’s study [24], and it is the BMI cut-off value for defining overweight recommended by the WHO [14], but this cut-off value may not be appropriate for Asians [25], because Asians have a higher body fat percentage than Caucasians at the same BMI level [26]. There are potential ethnic variations in body composition and build, and variations in the health risk associated with overweight among populations [14]. The cut-off level for overweight (24.0 kg/m^2^) has been developed using BMI for the general Chinese population by the WGOC [15,16,17], and according to the standard of WS/T 428–2013 (China), the optimal BMI cut-off value is 24 kg/m^2^ for Chinese adults. Whether the risk of incident hypertension of overweight subjects would decrease if they reduce their BMI to less than 24 kg/m^2^ and whether the risk of incident hypertension of subjects with BMI < 24 kg/m^2^ would increase if their BMI becomes higher than 24 kg/m^2^ should be examined.

We studied the relationship between five years’ change in BMI and the incidence of hypertension in 18- to 65-year-old Chinese adults with 3215 participants. In our study, out of 2120 individuals whose BMI was less than 24 kg/m^2^ at baseline, 415 persons developed overweight in 2011; out of 1095 overweight individuals at baseline, 170 persons were corrected to less than 24 kg/m^2^ in 2011. There was a significant trend of increased incidence of hypertension with an increase in the quartile of the BMI D-value in men (*p* for trend < 0.001) and women (*p* for trend = 0.004). During the five-year follow-up, the numbers of new cases of hypertension among people in the highest quartiles of the BMI D-value were different from those in the lowest quartiles in men and women (*p* < 0.05). The participants were further stratified by overweight status in 2006 and in 2011. Compared with non-overweight subjects in 2011, the ORs of incident hypertension were all significantly higher for overweight subjects, regardless of their overweight status at baseline (*p* < 0.05). These results indicate that the incident hypertension risk of overweight subjects would decrease if they reduce their BMI to less than 24 kg/m^2^ and that the incident hypertension risk of subjects with a BMI < 24 kg/m^2^ would increase if they become overweight.

Weight loss is a critical lifestyle modification for overweight and obese people to control hypertension. Although the exact mechanism of the effect of weight loss on BP is not clear, there are several plausible biologic pathways. First of all, the renin-angiotensin-aldosterone system is overactivated in obese subjects; meanwhile, aldosterone concentrations and renin activity are higher in obese subjects than in lean subjects [20]. Secondly, activity of the sympathetic nervous system is increased in hypertensive and obese subjects, and this might induce obesity-related renal effects. Alternatively, there may be inhibition of the natriuretic peptides system, of which the functional results are vasodilatation and natriuresis [20]. Thirdly, hyperinsulinemia and decreased insulin sensitivity as part of the metabolic syndrome may also form a necessary link between obesity and hypertension, although the interrelation is still not fully known [20].

Some limitations merit consideration in this study. First of all, data on lipid markers such as levels of low-density lipoprotein cholesterol and triglycerides, data on plasma glucose and data on family history of hypertension are not available in our study, so these variables were not adjusted in our analysis. Secondly, diet and physical activity are important factors for the incidence of hypertension, but these factors were not included in this analysis. Further studies are needed to include these factors in analyses. Thirdly, we cannot determine the time order between the change in BMI and the incidence of hypertension, and prospective studies are needed to determine the causal relationship between change in BMI and the incidence of hypertension. In addition, BMI, WC, WHR and WSR are four indices commonly used to measure the degree of obesity [4,5]. In some studies, WC was shown to be the best predictor of hypertension [27,28], and in some other studies, WSR was shown to be the best predictor of the incidence of hypertension [29,30]. Further studies should assess the association between change in these obesity indices and the incidence of hypertension.

## 5. Conclusions

In conclusion, the results from this study provide unequivocal evidence that weight loss makes a crucial contribution to the treatment of hypertension. Prevention of weight gain is likely to have a great impact on the incidence of hypertension in Chinese adults. Intervention programs designed to reduce BMI through lifestyle modification may have significant public health significance in reducing the risk of incident hypertension.

## Figures and Tables

**Table 1:** Baseline characteristics of 3215 study participants ^a^.

Variables	Men (*n* 1441)		Women (*n* 1774)	
	Non-Overweight Men (*n* 974)	Overweight Men (*n* 467)	Non-Overweight Women (*n* 1146)	Overweight Women (*n* 628)
Age (years)	45.1 ± 11.4	46.0 ± 10.2	44.6 ± 10.4	47.7 ± 9.1 ^b^
SBP (mm·Hg)	115.5 ± 10.5	119.4 ± 9.2 ^b^	110.8 ± 11.7	117.0 ± 10.4 ^b^
DBP (mm·Hg)	75.5 ± 7.3	78.8 ± 6.1 ^b^	72.7 ± 7.8	76.4 ± 7.1 ^b^
BMI (kg/m^2^)	21.2 ± 1.7	26.1 ± 1.8 ^b^	21.2 ± 1.8	26.4 ± 2.1 ^b^
Urban residence (%)	27.5	33.4 ^b^	27.8	29.5
Ever smoked cigarettes (%)	66.5	63.2	2.6	3.0
Alcohol drinker (%)	61.2	63.4	9.0	8.8
Incidence of hypertension (%)	16.6	32.1^b^	12.3	24.4 ^b^

Notes: SBP, systolic blood pressure; DBP, diastolic blood pressure; BMI, body mass index. ^a^ Values are means ± standard deviation or percentages; ^b^ Significantly greater odds, *p* < 0.05.

**Table 2:** The incidence of hypertension associated with BMI D-value in men and women.

Quartiles of BMI D-Value	Range (kg/m^2^)	Hypertension Cases (*n*)	No. of Subjects (*n*)	OR (95% CI)	
				Model 1 ^a^	Model 2 ^b^
Men (*n* 1441)					
Quartile 1	<−0.4783	75	360	1.000	1.000
Quartile 2	−0.4783–0.4920	77	360	1.460 (0.989–2.156)	1.448 (0.979–2.141)
Quartile 3	0.4921–1.6977	68	361	1.165 (0.783–1.731)	1.138 (0.764–1.696)
Quartile 4	≥1.6978	92	360	2.289 (1.552–3.376) ^c^	2.303 (1.560–3.401) ^c^
*p* for trend				<0.001	0.004
Women (*n* 1774)					
Quartile 1	<−0.5953	73	443	1.000	1.000
Quartile 2	−0.5953–0.4732	68	444	1.091 (0.741–1.606)	1.095 (0.743–1.613)
Quartile 3	0.4733–1.5054	66	444	1.157 (0.783–1.711)	1.152 (0.778–1.706)
Quartile 4	≥1.5055	87	443	1.755 (1.207–2.552) ^c^	1.745 (1.199–2.540) ^c^
*p* for trend				<0.001	0.004

Notes: BMI, body mass index; D-value, difference value between follow-up and baseline; OR, odds ratio; CI, confidence interval. ^a^ Logistic regression models were used, adjusting for age, BMI, SBP and DBP at baseline; ^b^ Logistic regression models were used, adjusting for variables in model 1 as well as smoking habits, alcohol consumption and place of residence at baseline; ^c^ Significantly greater odds, *p* < 0.05.

**Table 3:** The incidence of hypertension stratified by overweight status in 2006 and 2011.

Overweight Status in 2006	Overweight Status in 2011	Hypertension Cases (*n*)	No. of Subjects (*n*)	OR (95% CI)	
				Model 1 ^a^	Model 2 ^b^
Non-overweight	Non-overweight	228	1705	1.000	1.000
Non-overweight	Overweight	75	415	1.446 (1.067–1.961) ^c^	1.469 (1.082–1.992) ^c^
Overweight	Non-overweight	38	170	1.000	1.000
Overweight	Overweight	265	925	1.530 (1.023–2.288) ^c^	1.580 (1.053–2.370) ^c^

Notes: OR, odds ratio; CI, confidence interval. ^a^ Logistic regression models were used, adjusting for age, SBP and DBP at baseline; ^b^ Logistic regression models were used, adjusting for variables in model 1 as well as smoking habits, alcohol consumption and place of residence at baseline; ^c^ Significantly greater odds, *p* <0.05.

**Table 4:** The association between SBP D-value and DBP D-value and change in overweight status between 2006 and 2011.

Overweight Status in 2006	Overweight Status in 2011	SBP D-Value		DBP D-Value	
		Model 1 ^a^	Model 2 ^b^	Model 1	Model 2
Non-overweight	Non-overweight	Reference	Reference	Reference	Reference
Non-overweight	Overweight	2.980 (1.558–4.402) ^c^	2.933 (1.511–4.356) ^c^	2.552 (1.589–3.515) ^c^	2.528 (1.568–3.488) ^c^
Overweight	Non-overweight	Reference	Reference	Reference	Reference
Overweight	Overweight	3.152 (0.897–5.408) ^c^	3.285 (1.032–5.539) ^c^	2.419 (0.877–3.960) ^c^	2.550 (1.018–4.082) ^c^

Notes: SBP, systolic blood pressure; DBP, diastolic blood pressure; D-value, difference value between follow-up and baseline. ^a^ Multivariable linear regression models were used, adjusting for age, SBP and DBP at baseline; ^b^ Multivariable linear regression models were used, adjusting for variables in model 1 as well as smoking habits, alcohol consumption and place of residence at baseline; ^c^ *p* < 0.05.

## Abbreviations

The following abbreviations are used in this manuscript:

BMI: body mass index
CHNS: China Health and Nutrition Survey
OR: odds ratio
95% CI: 95% confident interval
D-value: difference value
DALYS: disability adjusted life years
WC: waist circumference
WHR: waist:hip ratio
WSR: waist:stature ratio
WHO: World Health Organization
WGOC: Working Group on Obesity in China
SBP: systolic blood pressure
DBP: diastolic blood pressure
SD: standard deviation
BP: blood pressure
